# Curvature-Adoptive CNC Machining of Freeform Optics via Dynamic Tangential Toolpath Optimization

**DOI:** 10.3390/ma18225153

**Published:** 2025-11-13

**Authors:** Ravi Pratap Singh, Yaolong Chen

**Affiliations:** Department of Mechanical Engineering, Xi’an Jiaotong University, 28 Xianning West Road, Xi’an 710049, China

**Keywords:** freeform optics, CNC grinding, toolpath optimization, CAD/CAM integration

## Abstract

The manufacturing of freeform optical lenses, essential for advanced applications such as Earth observation and laser fusion, demands exceptional surface accuracy and lightweight designs. However, their complex, non-symmetrical geometries present significant manufacturing challenges. Conventional CNC machining strategies, which rely on fixed Cartesian step sizes, are inherently inefficient for surfaces with rapidly varying curvature. This inadequacy results in non-uniform material removal, prolonged machining times, and substandard surface quality. This study presents a novel curvature-adaptive machining strategy based on dynamic tangential toolpath optimization. The method continuously aligns the toolpath with the local surface geometry to maintain uniform cutting conditions. A dedicated computer-aided manufacturing (CAM) software environment was developed to generate the optimized toolpaths and corresponding G-code. Experimental validation on representative freeform optics demonstrated a substantial improvement in precision: a single error-compensation iteration achieved a reduction in peak-to-valley form error of up to 48.4%. The results confirm that the proposed strategy significantly outperforms conventional fixed-step methods, delivering superior surface finish, reduced machining time, and enhanced process flexibility without requiring specialized hardware. This work establishes a practical and high-precision advancement for the manufacture of high-performance freeform optical systems.

## 1. Introduction

Freeform optical lenses have emerged as transformative elements in modern optical engineering, offering a level of design flexibility and performance unattainable with traditional spherical or aspherical components [1,2,3]. Unlike rotationally symmetric optics, freeform lenses can incorporate complex surface geometries that redistribute optical power in unconventional ways. This capability allows designers to correct higher-order aberrations, improve imaging performance, and integrate multiple optical functionalities into a single compact element. Their adoption spans a diverse range of high-performance fields, including Earth observation and remote sensing [4], augmented and virtual reality (AR/VR) systems [5], automotive LiDAR and advanced driver-assistance systems (ADASs) [6], high-power laser systems for fusion research [7], and military-grade imaging and targeting optics [8]. The recent push towards more compact and powerful systems in consumer electronics and telecommunications further underscores their critical role [9]. By reducing the number of optical elements required in a system, freeform lenses can decrease overall size and weight, improve energy efficiency, and enhance mechanical robustness factors that are critical for both portable consumer electronics and spaceborne platforms.

The unique capabilities of freeform optics stem directly from their departure from rotational symmetry. Spherical and aspherical lenses, although widely used, are constrained by their axisymmetric profiles, limiting their ability to correct complex field-dependent aberrations. In contrast, freeform surfaces can distribute optical power asymmetrically across the aperture, enabling wide fields of view, off-axis performance optimization, and compact packaging. For example, AR and VR devices increasingly rely on freeform components to deliver immersive visual experiences while maintaining small form factors that are comfortable for long-term wear [10]. Similarly, in automotive LiDAR systems, freeform optics provide precise beam shaping and scanning efficiency, improving detection accuracy and minimizing blind spots under dynamic conditions [11]. In space applications, weight reduction achieved through freeform designs translates directly into reduced launch costs and enhanced payload capacities.

However, the same characteristics that make freeform lenses appealing to optical designers present substantial challenges to manufacturing engineers. The surfaces of freeform optics exhibit highly variable slopes, curvatures, and local feature densities, making them significantly more difficult to fabricate than their spherical or aspherical counterparts [8,12]. Even minor deviations from the nominal profile can lead to measurable optical aberrations, degrading the performance of the entire optical system. Achieving the required sub-micron form accuracy and nanometer-scale surface roughness is therefore a central challenge in freeform optics fabrication [13].

Modern fabrication of freeform surfaces primarily relies on advanced computer numerical control (CNC) machining techniques such as deterministic microgrinding and sub-aperture polishing [14,15]. These processes remove material in controlled increments, progressively shaping the optical surface to its final form. While CNC machining offers the flexibility and precision needed for complex geometries, its effectiveness is strongly influenced by the toolpath generation strategy employed. Conventional toolpath planning methods, particularly fixed-step Cartesian approaches, move the cutting tool along evenly spaced intervals without regard to local surface features. This approach is effective for simple geometries, where curvature and slope vary gradually, but becomes inefficient and error-prone when applied to freeform optics [16]. Despite recent improvements in machine tool accuracy, the fundamental limitation of non-adaptive path planning remains a major bottleneck [17].

In fixed-step machining, the interaction between the cutting tool and the workpiece surface changes unpredictably as curvature varies. Regions of high curvature may experience excessive tool engagement, leading to overcutting, surface tearing, and accelerated tool wear [18]. Conversely, flatter regions may receive insufficient contact, resulting in incomplete material removal and surface waviness. These inconsistencies not only degrade surface quality but also extend machining cycles, as additional corrective passes are required. Over time, frequent tool changes, recalibrations, and machine downtime further inflate production costs [19,20]. For high-value optics, such as those used in space or defense, even small increases in fabrication inefficiency can have substantial economic impacts.

The dynamic forces exerted on the cutting tool during fixed-step machining also contribute to accelerated tool degradation. Variations in cutting angles and forces across the surface can induce chatter, microfractures, and local heating effects, reducing tool life and compromising machining accuracy [21]. As tool wear progresses, it introduces further variability into the machining process, creating a feedback loop that exacerbates surface errors. In precision applications where optical performance depends on form deviations well below one wavelength of light, these errors can render a component unusable. Effective management of tool wear is thus critical not only for maintaining consistent surface quality but also for ensuring the long-term reliability of the manufacturing process.

Metrology and quality control present additional challenges in freeform optics manufacturing. Traditional interferometric testing and contact-probe measurements are well-suited for spherical and aspherical components but struggle to characterize non-rotationally symmetric geometries with high accuracy [22,23]. Advanced techniques such as optical profilometry and high-resolution 3D surface mapping offer better fidelity for complex surfaces but are not trivial to integrate into the machining workflow. Real-time or in-process measurement is particularly difficult, yet it is essential for reducing rework cycles and ensuring that the fabricated surface conforms to design specifications. Bridging the gap between advanced metrology and adaptive machining strategies remains a critical area of development [24]. Recent efforts have focused on integrating on-machine probes and in situ interferometry [25,26], yet a seamless, automated loop for freeform optics is still lacking.

These difficulties highlight the need for a paradigm shift in CNC toolpath generation for freeform optics. Recent research has explored adaptive strategies that tailor machining parameters to local surface conditions, with promising results [27,28]. Adaptive control theory provides a robust theoretical framework for such strategies. By continuously monitoring process variables and adjusting machining parameters in real time, adaptive control can maintain stability and performance even in the presence of geometric or dynamic disturbances. When applied to toolpath planning, adaptive principles suggest aligning the cutter trajectory with the surface’s local tangential direction, a method known as tangential toolpath alignment. This approach ensures that the tool engages the surface consistently, minimizing abrupt changes in cutting dynamics and reducing the risk of surface defects [29].

Tangential alignment can be viewed as a form of real-time feedback control: by orienting the toolpath to match local curvature, the machining process becomes inherently more stable. Variations in cutting forces are distributed more evenly, tool wear is reduced, and the energy required for material removal is optimized. Additionally, by maintaining a consistent engagement angle, tangential alignment mitigates thermal and mechanical stresses on the workpiece, leading to improved dimensional stability and reduced subsurface damage [30]. In comparison with fixed-step approaches, tangential alignment has demonstrated substantial improvements in machining efficiency and surface finish in preliminary studies of complex geometries [31]. However, these approaches often lack a fully integrated digital thread from CAD to G-code and are not yet widely adopted in industrial settings [32].

Beyond the machining process itself, implementing tangential alignment requires an integrated software environment capable of translating surface geometry data into optimized CNC commands. This involves advanced mathematical modeling to calculate local tangents and curvatures, robust algorithms to generate smooth tool trajectories, and seamless integration with CAD/CAM platforms to ensure geometric fidelity. Simulation and validation modules are also critical for predicting potential issues such as tool interference, excessive force peaks, or machine kinematic limits. A user-friendly interface is essential to allow operators to input parameters, visualize toolpaths, and make adjustments without requiring deep expertise in computational geometry or control theory [33,34].

To address this critical gap, this work is founded on the central hypothesis that a dynamic, curvature-adaptive toolpath optimization strategy, which preemptively aligns the toolpath with local geometry, will fundamentally overcome the limitations of fixed-step methods. This hypothesis is investigated through the following specific research questions:

- Can the proposed curvature-adaptive algorithm yield a superior surface finish and higher form accuracy compared to conventional fixed-step methods?
- Can this strategy be effectively implemented through a dedicated, self-developed CAM software environment FreeForm-CAM (version 1.0) to create a practical and accessible solution?
- Will the integration of this software into a closed-loop process enable rapid convergence to sub-10 µm form accuracy within a single compensation iteration?

Consequently, this study presents a comprehensive curvature-adaptive CNC machining methodology for freeform optical lenses based on dynamic tangential toolpath optimization. A dedicated CAM environment, Freeform-CAM, was developed to implement this approach, combining the computational power of MATLAB (version R2025a) for mathematical modeling with the geometric accuracy of UG NX for surface representation. The system generates optimized toolpaths and G-code tailored to the local curvature of freeform surfaces. The proposed methodology was rigorously evaluated through experimental trials on representative optical components to test the posed hypotheses.

## 2. Freeform Surface Generation and Point Cloud Extraction

**Figure 1:**
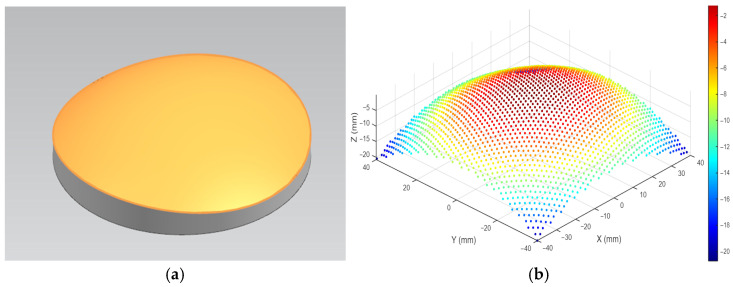
Schematic diagram of the freeform surface representation. (**a**) Continuous CAD model. (**b**) Discrete point cloud equivalent.

The successful implementation of any CNC machining strategy is contingent upon a high-fidelity digital representation of the target geometry. For freeform optical surfaces—complex, non-rotationally symmetric shapes defined by specific optical performance requirements—this foundation is a precise CAD model. This section details the process of creating the freeform surface within a commercial CAD environment and its subsequent conversion into a discrete point cloud. This point cloud serves as the fundamental input for the custom toolpath planning algorithms developed in this work. The transition from a continuous model to a discrete point cloud is illustrated in Figure 1.

### 2.1. CAD Modeling and Surface Definition in Siemens NX

The geometric foundation for this study was established using Siemens NX (vesrsion NX 12.0), a high-end CAD software. The freeform optical surfaces were designed directly within the NX environment using its advanced surface modeling tools. The process involved constructing a set of carefully defined non-uniform rational B-spline (NURBS) curves to frame the desired optical profile. These curves were then used as guides and boundaries to generate the final freeform surface, ensuring precise control over its shape, slope, and curvature distribution.

The resulting lens was modeled as a watertight solid body. Internally, Siemens NX represents such complex geometries using NURBS, which provide a mathematically exact and compact representation. A NURBS surface is defined by a control polygon net, associated weights, and knot vectors. The parametric formulation of a NURBS surface *S*(*u*,*v*), where *u*, *v* ∈ [0, 1], is given by:

(1) $$S(u,v)=\frac{\sum_{i=0}^{n}\sum_{j=0}^{m}N_{i,p}(u)N_{j,q}(v)w_{i,j}P_{i,j}}{\sum_{i=0}^{n}\sum_{j=0}^{m}N_{i,p}(u)N_{j,q}(v)w_{i,j}}$$

where *P_i_*_,*j*_ are the control points forming a bidirectional grid of size (*n* + 1) × (*m* + 1), *w_i_*_,*j*_ are the corresponding weights, providing additional influence over the surface’s shape. *N_i_*_,*p*_(*u*) and *N_j_*_,*q*_(*v*) are the B-spline basis functions of degrees *p* and *q*, defined on non-uniform knot vectors *U* and *V*, respectively.

The continuity and quality of the surface were validated using NX’s diagnostic tools, and the model was ensured to be free of gaps or flaws. The surface tolerance was maintained at a stringent value to ensure high geometric fidelity. This continuous NURBS model, an example of which is shown in Figure 1a, served as the master digital definition for all subsequent manufacturing steps.

### 2.2. Point Cloud Generation for CAM Processing

While the NURBS representation is ideal for design, the subsequent toolpath optimization algorithms, developed in MATLAB, operate on discrete data. Therefore, the continuous surface *S*(*u*,*v*) must be converted into a structured point cloud *P_k_*(*x_k_*,*y_k_*,*z_k_*). This is achieved through a systematic discretization process within NX. A uniform grid is defined in the 2D parametric domain:

(2) $$\begin{array}{l} u_{i}=\frac{i}{N_{u}},i=0,1,2,\ldots ,N_{u}\\ v_{i}=\frac{j}{N_{v}},j=0,1,2,\ldots ,N_{v} \end{array}$$

The corresponding 3D point cloud *C_i_*_,*j*_ is generated by evaluating the surface function at each parameter pair:

(3) $$C_{i,j}=S(u_{i},v_{j})$$

**Figure 2:**
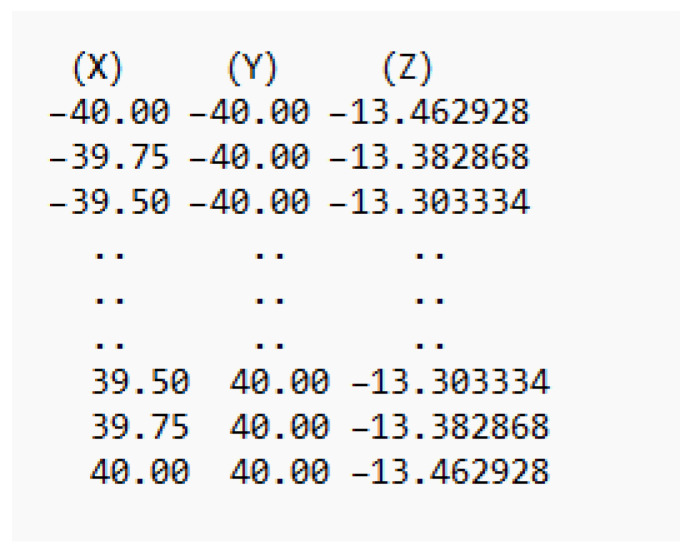
Sample of data file.

The spatial resolution of this point cloud is critical. The choice of sampling intervals $∆u=\frac{1}{N_{u}},\;and∆v=\frac{1}{N_{v}}\;$ must be fine enough to capture the highest spatial frequencies of the surface, adhering to a sampling criterion analogous to the Nyquist principle. The approximate Cartesian step size Δ*s* on the surface is related to the parametric steps by the metric of the first fundamental form:

(4) $$\Delta s\approx \sqrt{E{\left(\Delta u\right)}^{2}+2F\Delta u\Delta v+G{\left(\Delta v\right)}^{2}}$$

where, $\;E=\frac{\partial S}{\partial u}\cdot \frac{\partial S}{\partial u},F=\frac{\partial S}{\partial u}\cdot \frac{\partial S}{\partial v},G=\frac{\partial S}{\partial v}\cdot \frac{\partial S}{\partial v},\;$ are the coefficients of the first fundamental form. This ensures that the point cloud density is sufficient for accurate curvature calculation and toolpath generation. The final output was a structured point cloud, visualized in Figure 1b which was exported in a structured ASCII text format. A sample of this data file is illustrated in Figure 2, where each line contains the Cartesian coordinates (*X*, *Y*, *Z*) of a single surface point.

This file served as the primary input for a dedicated CAM software environment, named Freeform-CAM, which was developed in MATLAB for this research. The Freeform-CAM software imports this point cloud and internally calculates the required local geometric properties—such as unit normal vectors and surface curvature—to implement the dynamic tangential toolpath optimization strategy detailed in the following section. This integrated data pipeline from the CAD model to the custom CAM platform is fundamental to the proposed machining methodology.

### 2.3. Grinding Principles for Freeform Surfaces

**Figure 3:**
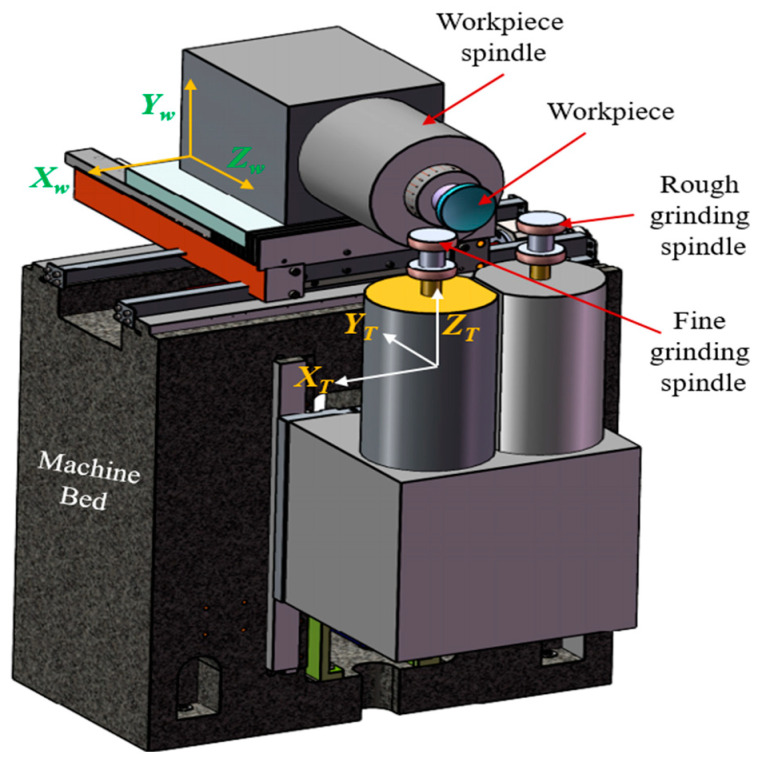
Schematic diagram of rough and fine grinding.

Grinding is a precision material removal process that utilizes abrasive particles bonded into a grinding wheel to remove material through a combination of plastic deformation and brittle fracture. When applied to freeform optical surfaces characterized by non-rotationally symmetric geometries and complex curvature variations, the grinding process must carefully balance material removal efficiency, surface finish, and geometric accuracy. Typically, a two-phase approach comprising rough grinding and fine grinding is employed to achieve the desired form accuracy and surface integrity. In the grinding process, material removal occurs through the interaction between individual abrasive grains and the workpiece surface, where each grain acts as a micro-scale cutting tool, removing a thin chip of material. On freeform surfaces, the non-uniform local curvature introduces additional challenges, influencing chip thickness, contact length, and force distribution, all of which must be carefully managed. The Material Removal Rate (*MRR*), a key performance metric in grinding, is defined as:

(5) $$MRR=v_{w}\cdot a_{p}\cdot v_{f}\cdot f$$

where *v_w_* is the workpiece velocity, *a_p_* is the depth of cut, *v_f_* is the feed rate, and f is the frequency of abrasive-grain engagement. For rough grinding, maximizing *MRR* is prioritized, while fine grinding focuses on minimizing surface roughness and subsurface damage. The schematic diagram of the rough and fine grinding setup, including the machine components and coordinate systems, is illustrated in Figure 3.

#### 2.3.1. Rough Grinding: Efficiency-Driven Material Removal

Rough grinding is the initial phase in the freeform surface machining process, aimed at rapidly removing the majority of excess material to approach the target geometry. This phase employs relatively coarse abrasive grits (typically in the range of 36–60 mesh) and aggressive machining parameters to achieve high Material Removal Rates (*MRR*s). While prioritizing efficiency, it is essential to maintain acceptable wheel wear and minimize detrimental workpiece damage.

The process is governed by significant tangential *Fₜ* and normal *Fₙ* forces exerted by the abrasive grains on the workpiece. These forces directly influence power consumption, wheel loading, and potential surface/subsurface damage. As highlighted in precision grinding and hard-turning studies, these forces can be approximated using Archard wear models and empirical force equations. On freeform surfaces, the varying local curvature alters the contact mechanics between the wheel and workpiece, leading to non-uniform force distribution. To address this, adaptive path planning is essential to maintain consistent engagement and avoid localized over-removal or form deviation. This phase sets the foundation for subsequent finishing, where the form accuracy and bulk material removal are optimized without compromising the structural integrity of the workpiece. The setup, including wheel and spindle configurations, is illustrated in Figure 3.

#### 2.3.2. Fine Grinding: Surface Finish and Accuracy Optimization

Fine grinding is the critical second phase focused on achieving high surface finish, precise form accuracy, and minimal subsurface damage. This phase utilizes much finer abrasive grits (typically 120–1000 mesh) and operates at reduced material removal rates compared to rough grinding. The emphasis shifts from bulk removal to ductile-regime cutting, where material is removed primarily through plastic deformation rather than brittle fracture, thereby significantly reducing the risk of micro-cracking and subsurface defects. During fine grinding, surface roughness (*Ra*) is influenced by grit size (*d*), feed rate (*f*), and workpiece hardness (*H*), as described by empirical models validated in precision optics manufacturing is given as,

(6) $$Ra\propto \frac{d^{0.8}\cdot f^{0.6}}{H^{0.4}}$$

This relationship underscores the importance of controlling feed rate and grit size to achieve the desired surface quality, especially in precision optics and high-end mechanical components. Furthermore, to achieve ultra-high surface finish, fine grinding employs low infeed rates and higher wheel speeds, which serve a dual purpose: Reducing chip thickness (*h*) to minimize cutting forces and thermal load. Enhancing thermal conductivity away from the contact zone, thereby preventing thermal damage such as burnishing, melting, or micro-cracking. The chip thickness (*h*) during fine grinding is given by

(7) $$h=\frac{v_{f}\cdot d}{v_{w}}\cdot {\left(\frac{1}{\pi }\right)}^{0.5}$$

For freeform surfaces, maintaining uniform chip thickness across regions of varying curvature is critically important. Uneven (*h*) values can lead to localized burning, tearing, or form errors. To address this, the present study proposes a dynamic toolpath orientation adjustment strategy, which ensures that (*h*) remains consistent even as the local surface curvature changes. This adaptive approach is key to achieving uniform surface quality and avoiding localized defects on complex freeform geometries.

## 3. Mathematical Framework for Local Curvature Estimation

The precision of a curvature-adaptive machining strategy is fundamentally governed by the accurate estimation of the local surface geometry across the freeform optic. Conventional toolpath planning, which relies on fixed Cartesian steps, fails to account for spatial variations in curvature, leading to non-uniform material removal. This study employs a differential geometry-based approach where the local morphology at any point is approximated by fitting a sphere to its immediate neighborhood. The radius of this sphere provides a stable, intuitive measure of local curvature, which directly dictates the optimal toolpath point density.

### 3.1. The Principle of Local Spherical Approximation

For a smooth, twice-differentiable surface, the local geometry at a point *P*_0_ = (*x*_0_,*y*_0_,*z*_0_) is characterized by its principal curvatures, *k*_1_ and *k*_2_ [35]. The mean curvature *H*, a fundamental property influencing material removal rates in sub-aperture is defined as their average:

(8) $$H=\frac{k_{1}+k_{2}}{2}$$

**Figure 4:**
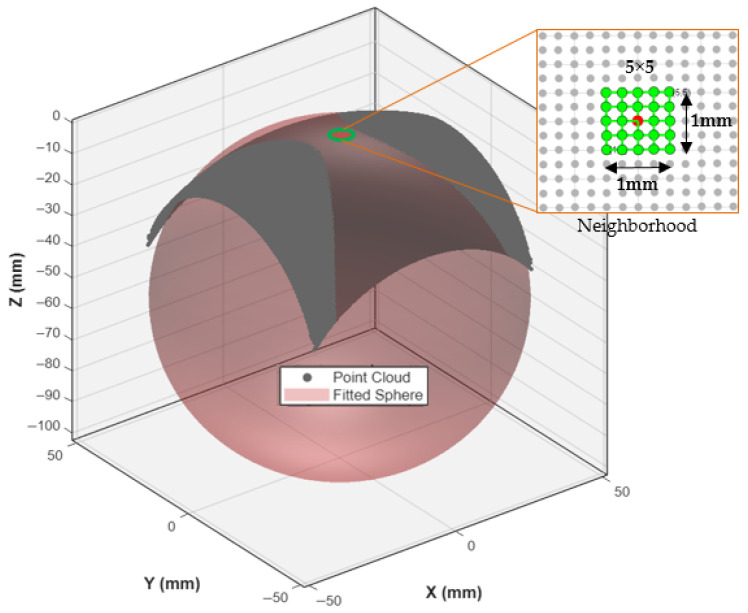
Schematic diagram of local curvature calculation via spherical approximation.

The corresponding mean radius of curvature (ROC) is given by *R* = 1/*H*. This work employs a robust numerical method to estimate R by fitting a sphere to a local neighborhood of points surrounding *P*_0_. in the surface point cloud. As illustrated in Figure 4, a 5 × 5 kernel of points is selected around each target point (shown in red). The radius R of the best-fit sphere to these 25 points provides a direct measure of the local curvature. This calculated radius, *R*, drives the dynamic path planning strategy. A small R indicates a highly curved region, necessitating a denser toolpath for accurate machining, while a large *R* signifies a flatter area where a sparser toolpath is sufficient. This principle of local spherical approximation forms the cornerstone of the adaptive algorithm.

A 5 × 5 neighborhood kernel is used to select points around the target location (red) on the freeform surface point cloud. The local morphology is approximated by fitting a sphere, the radius R of which provides the local radius of curvature.

### 3.2. Numerical Estimation via Least-Squares Sphere Fitting

Given a discrete point cloud *P_k_* = (*x_k_*, *y_k_*, *z_k_*) generated from the NURBS surface, the local curvature at a point is estimated from its local neighborhood. This implementation uses a (2*m* + 1) × (2*m* + 1) kernel with *m* = 2, resulting in a 5 × 5 kernel encompassing *N* = 25 points.

Let *N*(*i*,*j*) = *P_k_*∣*k* = 1, 2, …, *N* be the set of points in the kernel centered on the target point. The general equation of a sphere with center *O* = (*x_c_*, *y_c_*, *z_c_*) and radius *R* is:

(9) $${\left(x-x_{c}\right)}^{2}+{\left(y-y_{c}\right)}^{2}+{\left(z-z_{c}\right)}^{2}=R^{2}$$

This equation is expanded and rearranged into a linear form amenable to least-squares solution,

(10) $$x^{2}+y^{2}+z^{2}=2xx_{c}+2yy_{c}+2zz_{c}+\left(R^{2}-x_{c}^{2}-y_{c}^{2}-z_{c}^{2}\right)$$

By defining a new parameter vector u, this becomes a system of linear equations solvable via least-squares minimization:

(11) $$u={\left[2x_{c},2y_{c},2z_{c},R^{2}-x_{c}^{2}-y_{c}^{2}-z_{c}^{2}\right]}^{T}$$

The equation for each point *P_k_* is:

(12) $$x_{k}^{2}+y_{k}^{2}+z_{k}^{2}=\left[x_{k},y_{k},z_{k},1\right]\cdot u$$

For all *N* points, this forms the matrix system *Au* = *b*, where

$$A=\left[\begin{array}{cccc} x_{1} & y_{1} & z_{1} & 1\\ x_{2} & y_{2} & z_{2} & 1\\ \vdots & \vdots & \vdots & \vdots \\ x_{N} & y_{N} & z_{N} & 1 \end{array}\right],b=\left[\begin{array}{c} x_{1}^{2}+y_{1}^{2}+z_{1}^{2}\\ x_{2}^{2}+y_{2}^{2}+z_{2}^{2}\\ \vdots \\ x_{N}^{2}+y_{N}^{2}+z_{N}^{2} \end{array}\right]$$

The least-squares solution is given by,

(13) $$u={\left(A^{T}A\right)}^{-1}A^{T}b$$

The center *O* and radius *R* of the best-fit sphere are then recovered from the solution vector:

(14) $$x_{c}=\frac{u_{1}}{2},y_{c}=\frac{u_{2}}{2},z_{c}=\frac{u_{3}}{2},R=\sqrt{u_{1}^{2}+u_{2}^{2}+u_{3}^{2}+4u_{4}}$$

### 3.3. Enhancement: Aspect Ratio Correction for Anisotropic Surfaces

Freeform optics often exhibit significantly different scales in the *X* and *Y* directions (e.g., off-axis parabolic sections). A standard sphere fit on raw coordinate data can be biased if the point cloud is anisotropically sampled or if the surface patch itself is highly elongated.

To mitigate this, an aspect ratio correction is applied during the fitting process. The *Y*-coordinates are scaled by the global aspect ratio of the point cloud, *α* = *X_span_*/*Y_span_*, before constructing matrices *A* and *b*. The sphere is fitted to the scaled coordinates (*x_k_*,*αy_k_*,*z_k_*). The resulting radius of curvature *R* is the correct estimate for the original, unscaled geometry. This ensures the curvature estimation is isotropic and geometrically accurate, preventing artificial inflation of the radius estimate along the longer axis.

### 3.4. Quality Metric: Fit Error Estimation

The quality of the spherical fit is quantified by the root-mean-square (RMS) error εfit, which serves as a confidence metric for the curvature estimate:

(15) $$\varepsilon_{fit}=\sqrt{\frac{1}{N}\sum_{k=1}^{N}{\left(\left\|P_{k}-o\right\|-R\right)}^{2}}$$

A high *ε_fit_* indicates a region where the surface deviates significantly from a spherical shape (e.g., at an inflection point or a highly astigmatic region), flagging it for potential review or a more conservative toolpath strategy.

This robust numerical framework transforms the discrete point cloud into a continuous curvature field *k*(*i*,*j*) = 1/*R*(*i*,*j*), which is the primary input for the subsequent dynamic tangential toolpath optimization. This ensures material removal rates are precisely tailored to the local surface geometry, advancing the precision and efficiency of freeform optic manufacturing.

### 3.5. Determination of Optimal Tool Center Position Based on Local Surface Geometry and Curvature

**Figure 5:**
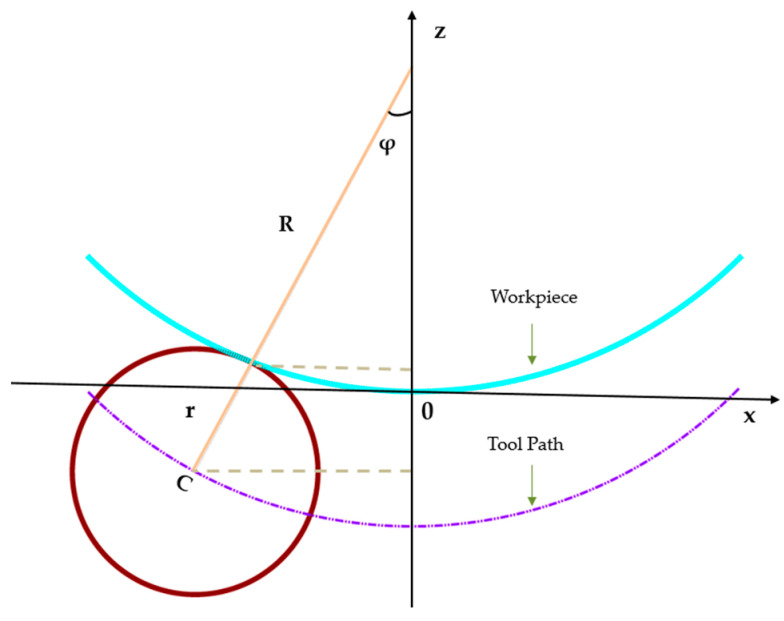
Schematic diagram of tool center point calculation.

Following the estimation of the local radius of curvature R at each discrete point (*C_i_*_,*j*_) on the freeform optical surface, the next critical step in curvature-adaptive toolpath planning is the precise determination of the Tool Center Position (*TCP*). The *TCP* defines the spatial location of the center of the spherical cutting or polishing tool such that it maintains tangential contact with the workpiece surface at the target point *C*, while conforming to the local curvature geometry. This section presents the mathematical derivation of the *TCP* location, beginning with the fundamental 2D engagement geometry between the tool and the workpiece surface is illustrated conceptually in Figure 5.

#### 3.5.1. Fundamental Geometry of Tool–Surface Engagement

As illustrated in Figure 5, the *TCP*, the contact point *C* on the workpiece, and the local curvature center *O* of the surface are collinear along the surface normal direction. This geometric constraint arises directly from the requirement of tangential contact between the spherical tool (of radius *R_t_*) and the freeform surface at point *C*.

The key geometric relationship is derived from the distance between the tool center (*TCP*) and the local curvature center *O*:

(16) $$\left\|TCP-O\right\|=\left|R-R_{t}\right|$$

where *R* is the local radius of curvature at point *C* (estimated via sphere fitting), *R_t_* is the radius of the spherical tool, $\hat{N}$ is the unit surface normal vector at *C*. The Tool Center Position (*TCP*) is thus expressed as a signed offset from the surface contact point *C* along the normal vector:

(17) $$TCP=C+D\cdot \hat{N}$$

The offset distance *D* depends on whether the surface is convex or concave. For a convex surface (*R* > 0),

(18) $$D=R_{t}-\left(R-\left\|C-O\right\|\right)$$

In the common simplification where *O* lies along the normal and the local surface is approximated as spherical, this reduces to *D* = *R_t_* − *R*. For a concave surface (*R* < 0),

(19) $$D=-R_{t}$$

Here, the tool center and curvature center lie on the same side of the surface contact point. This formulation ensures that the spherical tool maintains exact tangency with the freeform surface at the target point, adapting dynamically to local curvature variations.

#### 3.5.2. Radial Toolpath Alignment and Coordinate Transformation

In addition to the normal-direction offset, the tool center position must also be correctly positioned in the radial toolpath plane, particularly for rotational symmetry or radially varying toolpaths common in optical fabrication (e.g., lens grinding/polishing). As illustrated in Figure 5, let the radial distance from the optical axis (*Z*-axis) to the contact point *C* be:

(20) $$r=\sqrt{X^{2}+Y^{2}}$$

The angle *ϕ* between the surface normal and the *Z*-axis is derived as,

(21) $$\sin\phi =\frac{r}{R},\cos\phi =\sqrt{1-\sin^{2}\phi }$$

The effective radial distance from the optical axis to the tool center point (*TCP*) is then:

(22) $$R_{c}=(R+R_{t})\sin\phi$$

The angular position *θ* is preserved from the workpiece point to the tool center as,

(23) $$\begin{array}{l} TCP_{x}=R_{c}\cos\theta \\ TCP_{y}=R_{c}\sin\theta \\ TCP_{z}=Z+R_{t}\cos\phi \end{array}$$

This radial transformation ensures that the tool engagement is not only normal to the surface but also correctly aligned with the intended toolpath geometry, such as concentric or spiral paths often used in optical manufacturing.

#### 3.5.3. Enhanced Geometric Model for High-Precision Freeform Optics

For high-precision freeform surfaces such as aspheric, toric, or off-axis optics, the basic spherical engagement model must be extended to account for: Anisotropy in surface curvature (e.g., differing *X*-span and *Y*-span curvatures), higher-order geometric effects (e.g., astigmatism, irregular slope distributions), and non-uniform tool engagement across complex surface patches. To address these factors, an enhanced geometric model is introduced, incorporating: An aspect ratio correction factor $\;\alpha =\frac{X_{span}}{Y_{span}}$, Taylor-series or polynomial corrections for local surface deviations, Separate formulations for convex (*R* > 0) and concave (*R* < 0) geometries. The complete enhanced model yields the refined tool center position as:

(24) $$TCP=\left[\begin{array}{l} R_{c}\cos\theta \\ R_{c}\sin\theta /\alpha \\ Z+R_{t}\cdot f(\phi ,surface) \end{array}\right]$$

where the function *f*(*ϕ*,surface type) encapsulates higher-order offset corrections based on the local surface normal, curvature magnitude, and tool–surface engagement geometry.

This enhanced *TCP* model ensures sub-micron accuracy in tool placement—critical for ultra-precision finishing of freeform optical components where surface irregularities, edge effects, and anisotropic wear can significantly impact final quality.

### 3.6. Dynamic Tangential Spiral (DTS) Toolpath Generation for Freeform Surfaces

**Figure 6:**
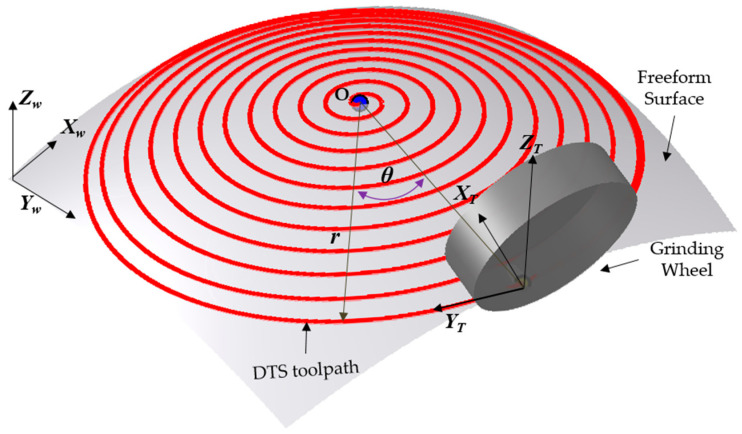
Schematic diagram of dynamic tangential toolpath.

The Dynamic Tangential Spiral (DTS) algorithm is a curvature-aware, 5-axis-compatible toolpath generation strategy designed to achieve high surface fidelity, minimal scallop height, and efficient material removal on complex freeform optical components. As illustrated in Figure 6, the DTS transforms a discrete Tool Centre Point (*TCP*) cloud into a smooth, continuous spiral trajectory that closely conforms to the local geometry of the workpiece. By integrating curvature-adaptive stepover, adaptive angular progression, and tangential B-axis alignment, the DTS method maintains a nearly constant scallop height, reduces lateral cutting forces, and avoids redundant or inefficient tool passes, outperforming conventional fixed-step Archimedean spirals or raster strategies.

#### 3.6.1. Toolpath Geometry and Parametric Formulation

As illustrated in Figure 6, the generated DTS toolpath is represented by a series of red concentric circles superimposed on the freeform surface. The spatial relationship between the toolpath and the workpiece is defined within both the workpiece coordinate system (*X_w_*, *Y_w_*, *Z_w_*) and the tool coordinate system (*X_t_*, *Y_t_*, *Z_t_*). The trajectory is parameterized in polar coordinates (*r*, *θ*), where *r*(*θ*) is the radial distance of the toolpath from the center, and *θ* is the angular position around the spiral center. The 3D position vector of the tool center at angle *θ* is expressed as:

(25) P(θ)=(X(θ),Y(θ),Z(θ))=(r(θ)cosθ,r(θ)sinθ,F(r(θ)cosθ,r(θ)sinθ))

where *F* defines the surface sag or elevation based on the projection of the spiral in the *XY* plane. To ensure uniform lateral spacing Δ*s* between adjacent toolpath revolutions a key factor in controlling scallop height, the radial distance *r*(*θ*) is adaptively adjusted via a curvature-sensitive contraction law:

(26) $$\frac{dr}{d\theta }=-\frac{\Delta s(k_{1},k_{2})}{2\pi }$$

Here, *k*_1_ and *k*_2_ are the local principal curvatures of the surface at point *X*(*θ*), *Y*(*θ*), and (Δ*s*) is the target stepover distance. Unlike the constant radial derivative in a classical Archimedean spiral *dr*/*dθ* = const, Equation (26) introduces real-time curvature feedback, causing the spiral to contract more tightly in high-curvature (convex) regions and remain more open in flatter zones. This adaptive behavior ensures more uniform surface engagement, minimizing scallop variations and improving edge definition.

#### 3.6.2. Curvature-Adaptive Stepover

The scallop height *h*, the vertical cusp between adjacent toolpaths is fundamentally governed by the stepover distance Δ*s* and the local radius of curvature *R* = 1/*k*:

(27) $$h=\frac{\Delta s^{2}k}{2}$$

To maintain a constant target scallop height *h_target_*, the nominal stepover is modulated by a curvature-dependent scaling factor:

(28) $$\Delta s(k_{1},k_{2})=\Delta s_{no\min al}\eta (k_{1},k_{2}),\eta (k_{1},k_{2})=1+\alpha (k_{1}+k_{2})$$

where Δ*s_nominal_* is the default stepover for flat regions, *α* is a tuning coefficient controlling sensitivity to curvature, (*k*_1_ + *k*_2_) represents the combined curvature magnitude. This formulation ensures that high-curvature regions receive smaller stepovers for finer surface finish, while flatter areas use larger stepovers to improve machining efficiency. Pre-smoothing of principal curvatures (e.g., via Gaussian filtering) is applied to avoid noise-induced instability in stepover modulation.

#### 3.6.3. Adaptive Angular Progression

A uniform angular increment (*dθ*) across the entire spiral can lead to oversampling near the center (where radial steps are small) and under sampling at the periphery (where the tool covers more area per revolution). To address this, DTS employs an adaptive angular spacing law:

(29) $$d\theta =\min(d\theta_{\max},d\theta_{\min}+\beta {\left(r_{transition}-r\right)}^{p})$$

where (*dθ*_min_) and (*dθ*_max_) define the minimum and maximum allowable angular steps, *r_transition_* is the radial transition point where angular density begins to decrease, *β* and *p* are tuning parameters controlling the rate of angular progression. This strategy ensures finer angular resolution in the central region for precision and uniformity.

#### 3.6.4. Tangential Alignment and B-Axis Orientation

In five-axis machining, maintaining the tool’s cutting face tangential to the spiral trajectory minimizes lateral forces, tool vibration, and wear. The instantaneous *B*-axis angle (tool rotation about the *Y*-axis) is derived directly from the spiral’s *XY* projection:

(30) $$B(\theta )=\arctan2\left(Y(\theta ),X\left(\theta \right)\right)$$

For regions with steep local slopes, the nominal *B*-axis orientation is refined by blending it with the local surface normal vector *n*(*θ*) = ∇*F*(*X*,*Y*), ensuring smoother engagement and better tool conformity. Additionally, to prevent tool gouging due to sudden changes in surface height, the local slope angle *ϕ* is monitored:

(31) $$\phi =\arctan\left(\frac{\left|Z_{i+1}-Z_{i}\right|}{\sqrt{{\left(X_{i+1}-X_{i}\right)}^{2}+{\left(Y_{i+1}-Y_{i}\right)}^{2}}}\right)$$

If *ϕ* > *ϕ*_max_ (a predefined threshold), intermediate toolpath nodes are inserted to ensure gradual height transitions, controlled material removal, and stable machining conditions.

### 3.7. Enhanced Error Compensation Strategy

Achieving sub-micron accuracy in freeform optical grinding requires a sophisticated strategy to correct for systematic errors inherent in the machining process. These errors, arising from tool deflection, wheel wear, thermal drift, and machine kinematics, cause deviations between the machined surface and the target design. This section details an enhanced, geometry-aware compensation framework that integrates metrology data with a model of the tool-surface interaction to generate a corrected toolpath.

#### 3.7.1. Error Mapping and Interpolation

The foundation of the compensation is the quantification of the machining error. After the initial grinding cycle, the workpiece is measured using a Hexagon high-precision Coordinate Measuring Machine (CMM) from Germany, producing a measured point cloud (*X_m_*,*Y_m_*,*Z_meas_*). The local machining error *E*(*X*,*Y*) at any measured point is defined as the deviation from the nominal design surface *Z_ideal_*(*X*,*Y*):

(32) $$E\left(X_{m},Y_{m}\right)=Z_{meas}\left(X_{m},Y_{m}\right)-Z_{ideal}(X,Y)$$

As the CMM data is discrete and may not cover the entire surface uniformly, this error must be interpolated to create a continuous error field *E_interp_*(*X*,*Y*) across the entire machining domain. This is achieved using a robust scattered data interpolation method (e.g., natural neighbor interpolation):

(33) $$E_{\mathrm{int}erp}\left(X,Y\right)=F_{\mathrm{int}erp}\left(X,Y;E\left(X_{m},Y_{m}\right)\right)$$

This continuous error map allows for the estimation of errors even at points not directly probed by the CMM.

#### 3.7.2. Geometric Correction for Tool-Surface Interaction

Simply subtracting the interpolated error is insufficient, as it does not account for the finite radius of the spherical grinding tool. The compensation must be adjusted based on the local surface geometry (convex or concave) to ensure the tool tip contacts the correct point on the surface. The initial corrected surface, obtained by removing the systematic error, is:

(34) $$Z_{corr}\left(X,Y\right)=Z_{ideal}\left(X,Y\right)+E_{\mathrm{int}erp}\left(X,Y\right)$$

However, the final toolpath must position the tool center point to achieve contact at *Z_corr_*. The required adjustment is a function of the tool radius *R_t_* and the local surface inclination angle *ϕ*, where

(35) $$\phi =\arctan\left(\sqrt{{\left(\partial Z/\partial X\right)}^{2}+{\left(\partial Z/\partial Y\right)}^{2}}\right)$$

For Convex Regions (*k* > 0): The tool center must be offset outward along the surface normal. The compensated *Z*-height for the toolpath is:

(36) $$Z_{comp}^{convex}\left(X,Y\right)=Z_{corr}\left(X,Y\right)+R_{t}\cdot \cos\phi$$

For Concave Regions (*k* < 0): The tool center must be offset inward. To account for potential anisotropic (elongated) geometries, an aspect ratio factor *A* (e.g., *A* = *X_span_*/*Y_span_*) is incorporated. The compensation model for concave zones is:

(37) $$Z_{comp}^{concave}\left(X,Y\right)=Z_{corr}\left(X,Y\right)-R_{t}\cdot \frac{\cos\phi }{A^{0.8}}$$

The exponent 0.8 was determined empirically to optimize compensation effectiveness without introducing new distortions in steeply curved areas.

#### 3.7.3. Compensation Validation and Final Toolpath Generation

The total local compensation applied at each point is quantified as the difference between the projected toolpath based on the measured surface and the newly calculated, compensated height:

(38) $$\Delta_{comp}\left(X,Y\right)=Z_{comp}\left(X,Y\right)-Z_{proj}\left(X,Y\right)$$

This value Δ*_comp_* serves as a key metric for validating the compensation, highlighting regions of significant adjustment. The final output of this strategy is a fully calibrated toolpath, which integrates the error correction with the geometric tool-offset compensation. This toolpath is then executed in a subsequent grinding cycle, resulting in a workpiece geometry that converges dramatically toward the target design, as demonstrated by the experimental results in Section 5.

### 3.8. Comparative Justification with Conventional CAM Methods

The sophisticated mathematical framework detailed in this chapter is fundamentally more complex than the standard toolpath generation algorithms embedded in commercial CAM software. This added complexity is necessitated by the stringent sub-micron form accuracy and uniform surface quality requirements for freeform optics, which conventional methods are inherently inefficient at achieving. The key distinctions are summarized in Table 1.

In essence, while a CAM programmer can generate a toolpath for a freeform surface using standard fixed-tolerance methods, the process is inherently inefficient and the resulting quality is not optimized. The programmer must often resort to manually partitioning the surface and applying different strategies, or using an excessively fine, globally fixed stepover to guard against errors in high-curvature zones. This leads to prohibitively long machining times and does not solve the core problem of variable cutting forces and tool wear.

The methodology presented in this work automates this high-level process planning. It translates the optical designer’s requirement for uniform, high-quality surface finish into a fundamental driver for the toolpath generation itself. The “execution tolerance” in our method is not a fixed geometric value but a dynamic parameter based on real-time local curvature. This ensures that machining effort is applied precisely where it is needed to achieve sub-micron precision without unnecessary overhead, thereby justifying the advanced mathematical framework.

## 4. Architecture of the Freeform CAM Software Development

**Figure 7:**
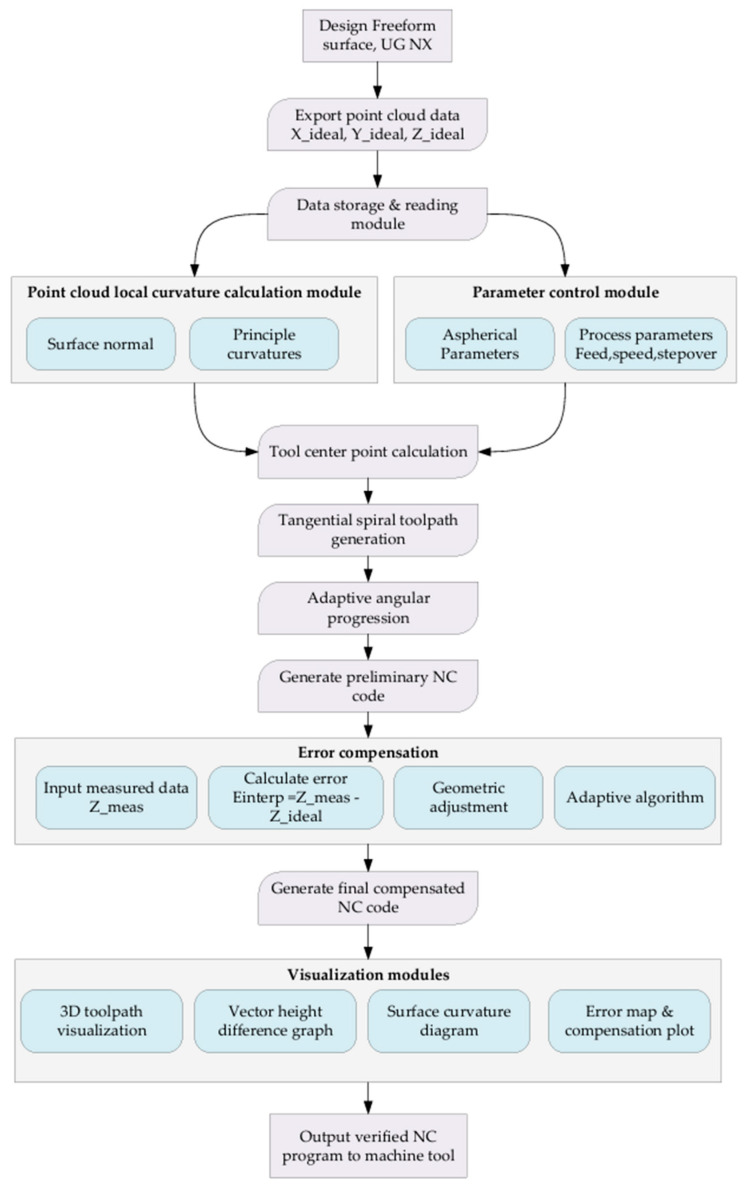
Schematic Architecture of Freeform CAM Software.

To overcome the limitations of conventional fixed-step CNC machining for freeform optics, a dedicated CAM framework was developed that integrates curvature-adaptive toolpath generation, real-time error compensation, and end-to-end process verification. The proposed architecture is illustrated in Figure 7, unifies four core functional modules—(1) design data import and storage, (2) curvature-aware toolpath planning, (3) adaptive error compensation, and (4) visualization and post processing—into a MATLAB-based software environment. Each module is engineered to preserve geometric fidelity while optimizing machining efficiency for non-rotationally symmetric freeform surfaces.

### 4.1. Design Data Import and Storage Module

Freeform optical geometries are initially defined in a feature-rich CAD environment (e.g., UG NX), where parametric surface models enable precise control over form and tolerance. To ensure compatibility with downstream machining workflows, the first stage of our CAM architecture exports idealized point-cloud data (*X*_ideal, *Y*_ideal, *Z*_ideal) directly from the CAD model. These discrete points serve as the foundation for all subsequent geometric computations. A custom-built data storage and retrieval subsystem then manages this point cloud, ensuring efficient access during curvature analysis, toolpath generation, and error mapping. By decoupling design from manufacturing via a standardized point-cloud interface, the framework preserves the integrity of the original freeform surface while enabling modular software development.

### 4.2. Curvature-Aware Toolpath Planning Module

The heart of the architecture lies in its ability to dynamically adapt toolpaths to local surface curvature. This module comprises two subcomponents: surface normal and principal curvature computation and tangential spiral toolpath generation. First, surface normal and principal curvatures are calculated at each discrete point in the imported cloud. These geometric descriptors quantify how the surface bends in the neighborhood of each point, enabling the software to distinguish regions of high versus low curvature. Next, a tangential spiral strategy aligns the tool center point (*TCP*) motion with the local principal directions. Unlike conventional rectilinear or circular interpolation, this approach ensures that the cutting or polishing tool moves tangentially to the surface gradient, thereby minimizing abrupt changes in material removal rate. Adaptive angular progression further refines the toolpath by adjusting stepover based on real-time curvature feedback—tightening spacing in high-curvature zones to maintain uniform stock removal and loosening it in flatter regions to reduce cycle time. The result is a smooth, curvature-responsive toolpath that balances precision and productivity.

### 4.3. Adaptive Error Compensation Module

Even with optimized toolpaths, geometric deviations between the designed surface (*Z*_*ideal*) and the machined workpiece (*Z*_*meas*) remain inevitable due to tool deflection, material inhomogeneity, or thermal effects. To close this loop, our architecture incorporates a physics-informed error compensation workflow. Measured surface data (captured via metrology such as coordinate measuring machines or interferometry) are first imported and interpolated to match the resolution of the original point cloud. A geometric adjustment algorithm then computes the signed error (*E*_*interp* = *Z*_*meas* − *Z*_*ideal*) at each measurement location. Rather than applying a global correction, an adaptive strategy distributes these corrections locally, respecting the kinematic limits of the CNC machine and the material removal characteristics of the process (e.g., grinding wheel wear, slurry flow in polishing). This closed-loop correction ensures that the final machined surface converges to the design intent with minimal iterative effort.

### 4.4. Visualization and Post Processing Module

To validate the toolpath and error correction workflows, the software includes a suite of visualization tools. A 3D toolpath renderer provides intuitive spatial context, while vector height difference plots and surface curvature diagrams highlight discrepancies between the ideal and compensated geometries. An error map and compensation plot further quantify the magnitude and distribution of corrections, enabling engineers to assess process stability before generating final G-code. Once validated, the compensated NC code is exported directly to the machine tool, completing the CAM-to-CNC pipeline.

## 5. Experimental Verification of CAM Software

**Figure 8:**
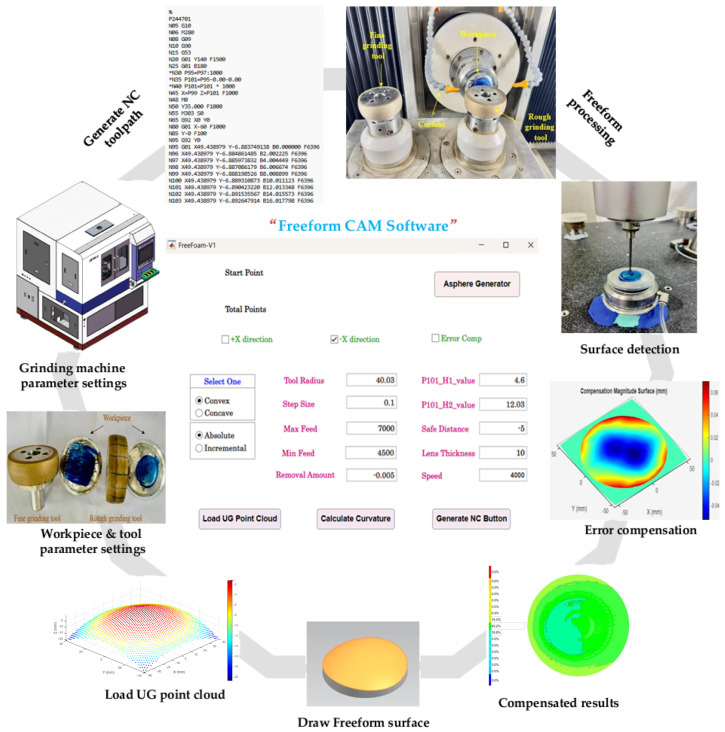
Experimental setup of closed-loop freeform manufacturing and error compensation with CAM software.

To validate the efficacy and precision of the Freeform CAM Software in a real-world manufacturing scenario, a comprehensive experimental setup was devised, centered around a closed-loop manufacturing process, as illustrated in Figure 8. The investigation utilized two distinct workpieces fabricated from H-K9L glass, selected for its relevant optical and mechanical properties: (i) 80.600FY and (ii) 850FY.

The process began with the design of each workpiece in UG NX. To mitigate the introduction of re-fixturing errors during the closed-loop process, a kinematic coupling principle was employed. A custom socket was machined into the workpiece base, which mates precisely with a corresponding fixture on the grinding machine table. This positive location scheme ensures highly repeatable positioning (estimated repeatability < 2 µm) each time the workpiece is unloaded for CMM measurement and reloaded for compensated grinding. The point cloud data defining the target geometry was subsequently exported directly to the Freeform CAM Software. Within the CAM environment, all necessary data processing and toolpath planning were conducted. Critical machining parameters were meticulously configured, including the grinding wheel radius (40.0125 mm), step size, feed rates, removal amount, and speed. The software then generated the Numerical Control (NC) code. This code was first validated through a digital simulation within the software to identify and rectify any potential errors, thereby minimizing the risk of machine damage or workpiece defects. The verified code was then transferred to the grinding machine for execution. Following the initial grinding operation, each workpiece was measured using a high-precision Coordinate Measuring Machine (CMM) from Hexagon Manufacturing Intelligence (Wendlingen, Germany). This step provided the essential metrological data, a high-fidelity 3D deviation map that fed into the core innovation of this process: the error compensation loop. When deviations from the target geometry were detected, the CAM software’s algorithm calculated a compensated NC code. This code accounted for the observed systematic and quasi-systematic errors, and the workpiece was re-ground. This closed-loop process of manufacture-measure-compensate-remanufacture was critical for achieving the final high-precision form.

### 5.1. Quantitative Analysis of Surface Form Error

**Figure 9:**
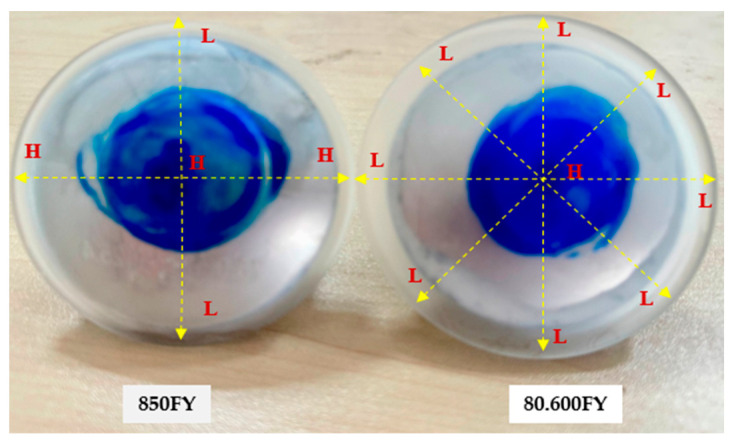
Freeform-machined workpiece.

The efficacy of the closed-loop manufacturing process was evaluated quantitatively using the Peak-to-Valley (PV) error, a critical metric that quantifies the total vertical distance between the highest peak and deepest valley on the surface error map. The two representative freeform optics used for this validation are illustrated in Figure 9. The 850FY workpiece exhibits a distinctly asymmetrical geometry, with a significant height differential between the low ‘*L*’ and high ‘*H*’ regions. In contrast, the 80.600FY workpiece features a nominally symmetrical shape, indicated by the marked region ‘*L’*, where the curvature is relatively uniform around the lens periphery. The quantitative results for both workpieces, measured before and after a single iteration of the error compensation cycle, are presented in Table 2 and discussed in detail below.

#### 5.1.1. Workpiece 850FY

**Figure 10:**
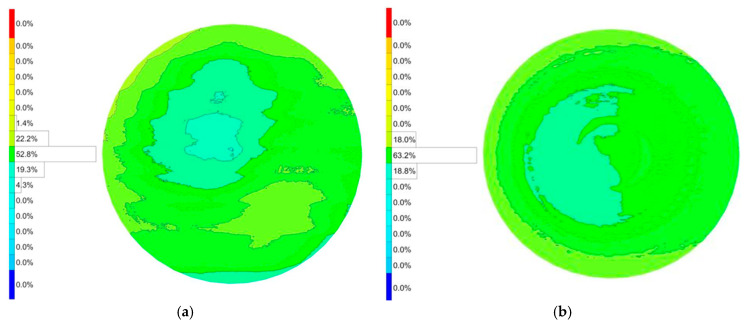
Surface error after freeform grinding. (**a**) Before error compensation. (**b**) After error compensation.

The 850FY geometry, with its distinct shape and radii, presented a greater initial challenge, exhibiting a larger initial PV error of 15.7 µm. The error was asymmetrical, with a deep valley of −8.7 µm and a peak of +7.0 µm. The application of the error compensation loop proved exceptionally effective for this part. The final measured PV error was dramatically reduced to 8.1 µm. This constitutes a 48.4% reduction in form error. The algorithm successfully corrected both peaks and valleys, bringing the maximum negative deviation to −3.6 µm (a 58.6% improvement) and the maximum positive deviation to +4.5 µm (a 35.7% improvement). The results for both workpieces demonstrate the robustness and consistency of the closed-loop compensation mechanism across different part geometries. The detailed measured surface error can be seen in Figure 10.

#### 5.1.2. Workpiece 80.600FY

**Figure 11:**
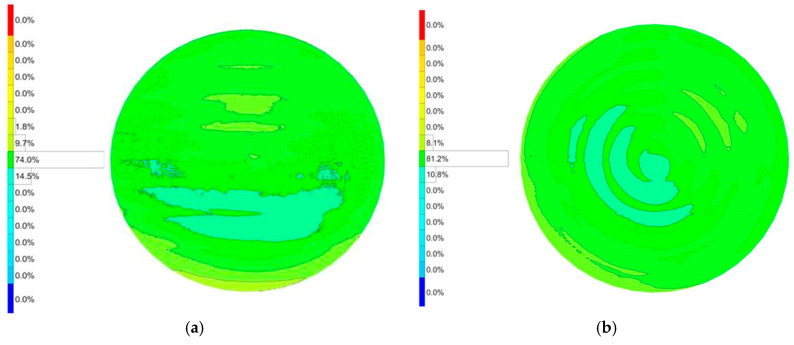
Surface error after freeform grinding. (**a**) Before error compensation. (**b**) After error compensation.

The initial grinding run for the 80.600FY workpiece yielded a surface with a PV error of 11.9 µm. The error profile was characterized by a maximum positive deviation (overcut) of +8.4 µm and a maximum negative deviation (undercut) of −3.5 µm. After a single iteration of the error compensation cycle, a significant improvement was observed. The PV error was reduced to 7.5 µm, representing a 37.0% improvement in overall form accuracy. The compensation algorithm was highly effective in mitigating both error extremes. The maximum overcut was reduced by 46.4% (from +8.4 µm to +4.5 µm), while the maximum undercut was also improved, decreasing by 14.3% (from −3.5 µm to −3.0 µm). The detailed measured surface error can be seen in Figure 11.

### 5.2. Comparative Discussion

The experimental results demonstrate the high performance of the proposed curvature-adaptive strategy. To contextualize these results, a comparison can be made with the state-of-the-art in freeform optics manufacturing. For instance, a study by František Procháska et al., 2023, on the precise grinding of optical free-form elements utilized a high-end commercial CAM software (Siemens NX) and a dedicated optical grinding machine (Satisloh SPM60) from Germany [36]. In their process, an initial conventional grind resulted in a form error of 15 µm PV, which was then reduced to 9 µm PV after applying a corrective grinding cycle.

The performance of our method compares favorably with this benchmark. Our proposed curvature-adaptive strategy, implemented in our custom-developed Freeform-CAM software, achieved a superior final form accuracy of 8.1 µm PV on a challenging asymmetrical workpiece (850FY), also within a single compensation iteration. Furthermore, our method achieved a more substantial relative error reduction (48.4%) from its initial state compared to the 40% reduction reported in the cited study.

This comparison highlights a key advantage of this approach. While the conventional method relied on a post-facto “corrective” step, our method is preemptive; the toolpath is dynamically optimized before machining based on local curvature to avoid non-uniform errors in the first place. The fact that this specialized algorithm can produce superior results to those achieved by established industrial methods underscores its significant potential.

## 6. Conclusions

This study was founded on the core hypothesis that a curvature-adaptive toolpath generation strategy, which dynamically aligns the toolpath with the local surface geometry, would significantly outperform conventional fixed-step methods in the machining of freeform optics. It was further hypothesized that this strategy could be implemented through a dedicated CAM software environment to create a robust and efficient closed-loop manufacturing process capable of achieving sub-10 µm form accuracy.

The experimental results provide compelling evidence to support these hypotheses. The proposed dynamic tangential toolpath optimization strategy, realized through the self-developed Freeform-CAM software, successfully addressed the inherent limitations of fixed-step methods. Validation on two distinct freeform workpieces (80.600FY and 850FY) confirmed a substantial improvement in precision, with a single compensation iteration achieving peak-to-valley (PV) error reductions of 37.0% and 48.4%, respectively, and final form accuracies below 8.1 µm. Furthermore, this performance was achieved without specialized hardware, demonstrating the software’s practical utility.

The findings lead to three principal conclusions that directly validate the initial hypotheses:

- The primary hypothesis is confirmed: The curvature-adaptive algorithm provides a mathematically sound and highly effective basis for toolpath generation. By maintaining uniform cutting conditions, it preemptively minimizes errors, resulting in superior surface quality and a faster convergence rate than the conventional, corrective approach embodied in commercial systems.
- The implementation hypothesis is validated: The successful integration of this algorithm into a custom CAM software proved critical. It offers a practical, hardware-agnostic solution that effectively bridges the gap between advanced theoretical algorithms and industrial applications.
- The process hypothesis is demonstrated: The synergistic closed-loop process, integrating intelligent toolpath generation with metrology-driven compensation, represents a significant leap in capability. It delivers a combination of high precision, improved efficiency, and enhanced process flexibility that is paramount for the manufacture of next-generation optical systems.

By delivering a practical, high-performance manufacturing solution, this work establishes a new paradigm for freeform optics fabrication. Future work will focus on extending the algorithm for five-axis machining and integrating real-time adaptive control to further enhance efficiency and unlock nanometer-level accuracies.

## Figures and Tables

**Table 1:** Comparison between conventional CAM and the proposed curvature-adaptive framework.

Feature	Conventional CAM Approach	Proposed Curvature-Adaptive Framework
Surface Approximation	Uses a single, user-defined tolerance for the entire surface. Complex areas are approximated with many small circular/spherical arcs, while simple areas may be over-sampled.	Employs a dynamic, physics-based metric (local radius of curvature) to dictate sampling density. The algorithm automatically concentrates computational effort where it is needed most.
Toolpath Strategy	Relies on fixed Cartesian or angular stepovers. The scallop height and material removal rate vary significantly across the surface, leading to non-uniform quality.	Uses a curvature-adaptive stepover (Equations (27) and (28)) to maintain a near-constant scallop height. This ensures uniform surface quality and prevents over- or under-machining.
Point Cloud Handling	Often involves “cloud healing” or smoothing as a pre-processing step, which can average out critical high-frequency form details essential for optical performance.	Uses the raw point cloud to calculate true local geometry. This method is a “smart” adaptive process, not simple smoothing, thereby preserving critical form details while optimizing the toolpath.
Primary Goal	Geometric Faceting: To create a toolpath that lies within a global tolerance zone of the CAD model. It prioritizes programming speed and generality.	Physical process optimization: To create a toolpath that produces a physically superior surface by maintaining constant cutting conditions. It prioritizes final surface precision and efficiency.
Resulting Precision	Effective for micron-level tolerances typical in mechanical components. Struggles to achieve sub-micron form accuracy efficiently due to its non-adaptive nature.	Designed specifically for sub-micron level form accuracy required in high-performance optics, as demonstrated by the experimental results in Section 5.

**Table 2:** Quantitative analysis of surface form error before and after CAM software error compensation.

Workpiece	Condition	Min Deviation (mm)	Max Deviation (mm)	PV (mm)
850FY	Before compensation	−0.0087	+0.0070	0.157
	After compensation	−0.0036	+0.0045	0.0081
80.600FY	Before compensation	−0.0035	+0.0084	0.0119
	After compensation	−0.0030	+0.0045	0.0075

## Data Availability

The original contributions presented in this study are included in the article. Further inquiries can be directed to the corresponding authors. However, the underlying software/code are not publicly available due to commercial confidentiality.
